# The eukaryotic translation initiation factor 3f (eIF3f) interacts physically with the alpha 1B-adrenergic receptor and stimulates adrenoceptor activity

**DOI:** 10.1186/s12858-015-0054-5

**Published:** 2015-10-23

**Authors:** Mario Javier Gutiérrez-Fernández, Ana Edith Higareda-Mendoza, César Adrián Gómez-Correa, Marco Aurelio Pardo-Galván

**Affiliations:** Instituto de Investigaciones Químico-Biológicas, Universidad Michoacana de San Nicolás de Hidalgo, Edificio B-3 Ciudad Universitaria Avenida Francisco J. Múgica S/N, Morelia, Michoacán 58030 México; Present address: Universidad Tecnológica de Morelia, Morelia, Michoacán 58200 México; División de Estudios de Posgrado de la Facultad de Ciencias Médicas y Biológicas “Dr. Ignacio Chávez”, Universidad Michoacana de San Nicolás de Hidalgo, Morelia, Michoacán 58020 México

**Keywords:** eIF3f, Alpha 1B-ADR, Gαq/11, Adrenoceptors, Protein-protein interaction

## Abstract

**Background:**

eIF3f is a multifunctional protein capable of interacting with proteins involved in different cellular processes, such as protein synthesis, DNA repair, and viral mRNA edition. In human cells, eIF3f is related to cell cycle and proliferation, and its deregulation compromises cell viability.

**Results:**

We here report that, in native conditions, eIF3f physically interacts with the alpha 1B-adrenergic receptor, a plasma membrane protein considered as a proto-oncogene, and involved in vasoconstriction and cell proliferation. The complex formed by eIF3f and alpha 1B-ADR was found in human and mouse cell lines. Upon catecholamine stimulation, eIF3f promotes adrenoceptor activity *in vitro*, independently of the eIF3f proline- and alanine-rich N-terminal region.

**Conclusions:**

The eIF3f/alpha adrenergic receptor interaction opens new insights regarding adrenoceptor-related transduction pathways and proliferation control in human cells. The eIf3f/alpha 1B-ADR complex is found in mammals and is not tissue specific.

## Background

The eukaryotic translation initiation factor 3f (eIF3f) is an ancient and conserved gene reported to be present in most eukaryotic organisms studied so far [1]. It was originally identified as a subunit of the protein synthesis-related eIF3 complex [2], where it is suggested to function as a protein synthesis inhibitor [3, 4]. In contrast, eIF3f acts as a translational enhancer by increasing protein synthesis efficiency in muscle hypertrophy [5]. Shut-off experiments in *Schizosaccharomyces pombe* showed that in a long-term period, eIF3f is essential for viability, and that depleting the expression of this gene markedly decreases global protein synthesis [6]. In accordance to this, cell viability was also compromised when eIF3f expression was decreased in proliferating human A549 cells [7].

The eIF3f protein is a member of the Mov-34 family. Members of this family contain an MPN (Mpr1/Pad N-terminal) motif, which is found in subunits of other macromolecular complexes such as the proteasome and the COP9 signalosome; it has been related to complex assembly promotion and to mediating protein-protein interactions [8–10]. The MPN domain of human eIF3f protein is flanked by a proline- and alanine-rich N-terminal region and by the C-terminal region.

During terminal muscle differentiation, eIF3f interacts with hypophosphorylated S6K1 through its MPN domain and with the mTOR/Raptor complex (mTORC1) by interacting with a TOS site contained in its C-terminal region [11]. As a consequence of this interaction, mTOR/Raptor phosphorylates S6K1, and thus regulates downstream effectors of mTOR and cap-dependent translation initiation [5, 11].

eIF3f shows a remarkable ability to interact with many other proteins involved in a variety of cellular functions which are not directly involved in protein synthesis. For instance, it has been reported that eIF3f participates in the deubiquitination and activation of the development-related transmembrane protein Notch 1 [12]. In addition, it has been shown that HIV-1 replication is inhibited by eIF3f, through its proline- and alanine-rich N-terminal region [13, 14]. This inhibition was observed with the full-length eIF3f protein and with the 91 amino acid N-terminal region of the protein. In both cases, HIV-1 mRNA levels were reduced as a result of eIF3f interfering with the HIV-1 mRNA 3’-end processing [13, 14]. Furthermore, a recent report indicates that eIF3f is capable of interacting with the DNA repair-related protein hMSH4, facilitating hMSH4 stabilization [15]. These authors demonstrate that the eIF3f-hMSH4 interaction is through the N-terminal regions of both proteins.

Considering the ubiquitous nature of eIF3f, the aim of this work was to investigate other stable eIF3f - protein interactions in native cellular conditions. In this study, we found a novel physical interaction between human eIF3f and the alpha 1B-adrenergic receptor (alpha 1B-ADR) and that eIF3f stimulates adrenoceptor activity.

## Materials and methods

### Chemicals and materials

All chemicals were purchased from Sigma Aldrich [St. Louis, MO, USA], unless otherwise noted. Materials were mainly purchased from Corning [Corning, NY, USA], Bio-Rad [Hercules, CA, USA], EMD Millipore [Billerica, MA, USA], and Eppendorf [Hauppauge, NY, USA], unless otherwise noted.

### Cell cultures

All cell lines were purchased from the American Type Culture Collection [ATCC, Manassas, VA, USA]. Human lung carcinoma A549 cells (ATCC CCL185), human hepatocellular carcinoma HepG2 cells (ATCC HB-8065), Burkitt’s Lymphoma Ramos cells (ATCC CRL-1596), and murine pre-osteoblast MC3T3-E1 Subclone 4 cells (ATCC CRL-2593) were thawed every month and routinely passaged twice per week into 75 cm^2^ flasks (Corning, Corning, NY, USA) to maintain them in a logarithmic growth phase at 37 °C in a humidified atmosphere with 5 % CO_2_ (NuAire, Plymouth, MN, USA). A549, HepG2, and Ramos were cultured in MEM medium supplemented with 10 % heat-inactivated FBS (Invitrogen, Carlsbad, CA, USA), 2 mM glutamine, 10 mM HEPES, and 1.5 g/L sodium bicarbonate. MC3T3-E1 cells were cultured in MEMalpha medium (Invitrogen) without ascorbic acid, supplemented with 10 % heat-inactivated FBS. At 85 % confluence, cells were harvested using 0.25 % Trypsin-EDTA solution and were sub-cultured or collected for subsequent experimental analysis. Since this study does not involve humans, human data or animals, an ethics committee approval was not required.

### Native western blot analysis

Cells were lysed with ProteoJET Mammalian Cell Lysis Reagent (Fermentas, Hanover, MD, USA), following the manufacturer’s protocol, to obtain total native conformation proteins. Protein concentrations were determined using a Bio-Rad Protein Assay Kit. A molecular weight (kDa) standard (NativeMark - Novex, Life Technologies, Grand Island, NY, USA) and equal amounts of protein extracts (50 μg) were subjected to electrophoresis on a 8 % SDS-free polyacrylamide gel (PAGE) and electrophoretically transferred to a polyvinylidene difluoride (PVDF) membrane following the manufacturer’s instructions (Bio-Rad, Hercules, CA, USA). After blocking non-specific binding sites with 5 % skimmed milk, blots were incubated with primary rabbit polyclonal antibody specific to eIF3f (Biolegend, San Diego, CA, USA) or primary goat antibody specific to alpha 1B-ADR (Santa Cruz Biotechnology, Santa Cruz, CA, USA), and horseradish peroxidase-conjugated goat anti-rabbit (Biolegend) or rabbit anti-goat secondary antibody (Santa Cruz Biotechnology). The bound antibody was detected by enhanced chemiluminescence (ECL) on an X-ray film (GE Healthcare Life Sciences, Piscataway, NJ, USA).

### Complex member identification

After native electrophoresis, the 120 kDa region of the gel was excised and eluted overnight at 4 °C in 1 mL of ProteoJet Mammalian Cell Lysis Reagent. For non-specific bound protein removal, the elution was incubated 1 h at 4 °C with a non-specific IgG and 100 μL of 50 % Protein A-Sepharose 4B beads (Invitrogen). After centrifugation, the supernatant was incubated at 4 °C with the antibody against eIF3f and 100 μL of 50 % Protein A-Sepharose 4B beads, washed 4 times with lysis buffer, resuspended in sample buffer (2 % SDS, 20 % glycerol, and 0.5 % bromophenol blue in 62 mM Tris HCl buffer, pH 6.8), boiled for 5 min, and subjected to electrophoresis on a 10 % SDS-polyacrylamide gel (10 % SDS-PAGE). A PageRuler Plus Prestained Protein Ladder (Thermo Scientific, Rockford, IL, USA) was included in all SDS-PAGEs. The resolved proteins were detected by Coomassie blue R250 staining (Bio-Rad); the unknown protein bands were excised and sent for N-terminal protein/peptide sequencing (Iowa State University of Science and Technology, Ames, IA, USA). After sequencing service, the partial amino acid sequences were subjected to a NCBI Blastp search to identify possible protein partners of eIF3f.

### Immunoprecipitate western blot analyses

As describe above, after a native electrophoresis, the 120 kDa region of the gel was excised, eluted, cleared with a non-specific IgG and Protein A-Sepharose 4B beads and centrifuged. The supernatant was incubated with the corresponding primary antibody (anti-eIF3f or anti-alpha 1B-ADR) and Protein A-Sepharose 4B beads, washed, resuspended in sample buffer, boiled, and subjected to electrophoresis (10 % SDS-PAGE). Proteins from the gel were electrophoretically transferred to a PVDF membrane and blotted with anti-eIF3f or anti-alpha 1B-ADR antibodies, and the corresponding secondary horseradish peroxidase-conjugated antibodies. The bound antibody was detected by ECL on an X-ray film.

### Plasmids

To express human eIF3f, we used the previously described pSK11F plasmid [7]. To express alpha 1B-ADR, we used plasmid AR0A1B0000 (Missouri S&T cDNA Resource Center, Rolla, MO, USA). To obtain plasmid eIF3fΔ91 (eIF3f lacking first 91 AA of the N-terminal region), template pSK11F and the forward 5’-CCCTTCCCCGGCGGCAGCATGGTC-3’ and reverse 5’-CAGGTTTACAAGTTTTTCATTG-3’ oligonucleotides were used to amplify the eIF3f coding sequence corresponding to amino acids 92–357. The forward oligo was designed to contain a classic Kozak consensus sequence (see underlined nucleotides), by modifying only 2 nucleotides. The amplicon was cloned in pGEM vector (Promega, Fitchburg, WI, USA) and verified by DNA sequencing (Elim Biopharmaceuticals, Hayward, CA, USA).

### [gamma-32P]GTP Binding Assay

Membranes from A549 human cells were obtained using ProteoExtract Subcellular Proteome Extraction Kit (Calbiochem, La Jolla, CA, USA), as described by the manufacturer. To obtain membranes with over expressed alpha 1B-ADR, before membrane extraction, cells were transiently transfected (48 h) with plasmid AR0A1B0000 using LipofectAMINE 2000 (Invitrogen) according to the manufacturer’s specifications and as described previously [7]. For in vitro translation of eIF3f and eIF3fΔ91, mRNA was synthesized *in vitro* using T3 and T7 RNA polymerase (Invitrogen), respectively, and according to the manufacturer’s instructions. The respective proteins were synthesized *in vitro* with a Rabbit Reticulocyte Lysate System (Promega), using 1 μg of mRNA. For the [gamma-32P]GTP binding assay [16], 20 μg of membrane protein and 5 μL of the translation reaction were resuspended in 55 μL of 50 mM Tris–HCl (pH 7.4), 2 mM EDTA, 100 mM NaCl, 1 μM GDP, 3 mM MgCl and 30 nM the [gamma-32P]GTP (Institute of Isotopes Co Ltd, Budapest, Hungary). As a control, the reaction was also performed using water instead of a mRNA. The reactions were incubated at 30 °C for 5 min in the presence or absence of agonist (100 nM adrenaline). The reaction was terminated by adding 600 μL of ice-cold stop solution (50 mM Tris–HCl pH 7.5, 20 mM MgCl2, 150 mM NaCl, 0.5 % Nonidet, 100 μM GDP, and 100 μM GTP) and incubating for 30 min in ice. To each reaction, non-specific IgG and 100 μl of Protein A-Sepharose 4B beads were added and further incubated on ice for 20 min. Non-specifically bound protein was removed by centrifugation. The supernatant was then incubated 1 h at 4 °C with 1 μg of Gαq/11 antibody (Santa Cruz Biotechnology) and immunoprecipitated with 100 μl of Protein A-Sepharose 4B beads for 1 h at 4 °C. Immunoprecipitates were collected, washed 4 times in buffer without detergent, and resuspended in a TE buffer. The samples were analyzed in a scintillation counter (Wallac, Oy, Turku, Finland).

### Statistical analysis

All experiments were independently repeated at least three times. Results of multiple experiments are expressed as mean ± standard error (S.E.). Analysis of Student’s t test was used to assess the differences between means. A *p* < 0.05 was accepted as statistically significant.

## Results

### eIF3f is immunodetected in a protein complex of approximately 120 kDa in native electrophoretic conditions

**Fig. 1:**
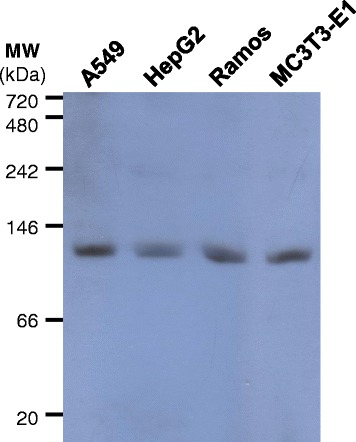
eIF3f is immunodetected in a protein complex of approximately 120 kDa in native conditions. Cells under exponential growth were lysed in a native-permissive buffer and 50 μg of total protein were subjected to electrophoresis in native 8 % polyacrilamide gels. Western blots were performed with an anti-eIF3f antibody (MW: protein molecular weight standard). Immunodetected protein complex in human A549 cells, a human lung carcinoma epithelial cell line; HepG2, a human hepatocellular carcinoma cell line; Ramos, a human Burkitt’s lymphoma cell line; and in murine MC3T3-E1 pre-osteoblast cells. A single prominent band of approximately 120 kDa is evident in all cell types

In denaturing electrophoresis conditions, mammalian eIF3f shows an apparent electrophoretic mobility of 47 kDa, albeit its molecular mass deduced from its amino acid sequence is 38.5 kDa. eIF3f migrates anomalously in SDS-PAGE, possibly due to the high proline content in the N-terminal region [2]. Human A549, HepG2, and Ramos, as well as murine pre-osteoblasts (cell line MC3T3-E1 Subclone 4), were independently lysed with a buffer that respects native protein conformation, as well as protein-protein interactions. Total protein from each cell type was subjected to a SDS-free PAGE and immunoblotted with eIF3f antibody. Figure 1 shows one major immunodetected band, of approximately 120 kDa, in each cell type. Other bands were observed in over-exposed blots (data not shown) and slightly appear in the blot shown in Fig. 1, around the 150 and 250 kDa region. The lower band intensity of these other complexes may be due to lower concentration, complex instability, or a transitory event. The eIF3 complex remained in the stacking gel. We tested several cell types to rule out the possibility that this immunodetected band may correspond to a specific cell line or organism. These results suggest that the 120 kDa complex may be present in mammalian organisms, and is not tissue specific.

### The 120 kDa complex is composed by eIF3f and the alpha 1B-adrenergic receptor

**Fig. 2:**
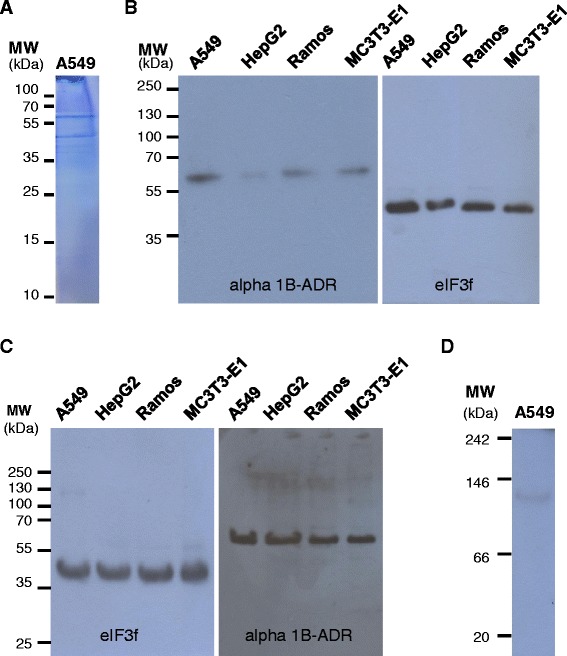
The 120 kDa complex is composed by eIF3f and the alpha-1B adrenergic receptor. **a** Resolved proteins in a SDS-PAGE from anti-eIF3f immunoprecipitate of the 120 kDa region: the expected 47 kDa corresponding to eIF3f, and an unknown ~60 kDa protein. The ~60 kDa protein was sent for N-terminal sequencing and a Blastp showed that it could be the alpha 1B-ADR. MW, prestained protein ladder; A549, resolved proteins in the 120 kDa complex obtained from total native protein extracts. **b** The 120 kDa complex was immunoprecipitated in native conditions with anti-eIF3f, and immunodetected with anti-alpha 1B-ADR or anti-eIF3f in denaturing conditions. **c** The 120 kDa complex was immunoprecipitated in native conditions with anti-alpha 1B-ADR, and immunodetected with anti-eIF3f or anti-alpha 1B-ADR in denaturing conditions. **d** Immunodetection of alpha 1B-ADR in native conditions using A549 cell extracts (MW: native protein standard), where the 120 kDa band is present. Results show that alpha 1B-ADR is the eIF3f partner in the 120 kDa complex

To identify the protein partners in the 120 kDa complex, total protein extracts under native conditions were used. Each of the 120 kDa regions were excised directly from the native gel, eluted in a protein-protein interaction permissive buffer, and immunoprecipitated with a specific antibody against eIF3f. After exhausting washes to eliminate unspecific protein binding, the immunoprecipitates were eluted in a non-reducing buffer, and run in a SDS-polyacrylamide gel. For proteins extracts from A549, Fig. 2a shows two eluted proteins, a 47 kDa protein (eIF3f) and a second protein of approximately 60 kDa; similar results were obtained with the other cell lines (data not shown). The 60 kDa protein resolved from each cell type was excised from the denaturing gel and sent to N-terminal sequencing service. The partial amino acid sequence [MNPDLDT] (which was the same in A549, HepG2, Ramos, and MC3T3-E1), its protein position, and the expected molecular weight were the criteria used in a NCBI Blastp search. Results of this search showed that the human protein that fulfilled these criteria was the alpha-1B adrenergic receptor (alpha 1B-ADR), a 56.836 kDa (520 amino acids) plasma membrane protein. To verify this, the 120 kDa complex was immunoprecipitated with eIF3f antibody and then immunodetected using an alpha 1B-ADR antibody and with eIF3f antibody (Fig. 2b). In addition, the 120 kDa complex was immunoprecipitated with alpha 1B-ADR antibody and then immunodetected with eIF3f antibody and alpha 1B-ADR antibody (Fig. 2c). Both assays were positive. These results show that alpha 1B-ADR physically and stably interacts with eIF3f. Furthermore, under native conditions the 120 kDa complex from A549 cell extracts was immunodetected with alpha 1B-ADR antibody (Fig. 2d), confirming that this complex is composed by eIF3f and alpha 1B-ADR.

### eIF3f promotes adrenoceptor activity upon catecholamine stimulation

The stable interaction between eIF3f and alpha 1B-ADR raised the question if this interaction had a functional consequence. That is, if eIF3f stimulates or inhibits Alpha 1B-ADR activation. Alpha 1B-ADR is a member of the G protein-coupled receptor family of alpha 1-adrenergic receptors (alpha 1-ADRs), which is composed by the subtypes 1A, 1B, and 1D; all of which signal through the Gq/11 family of heterotrimeric G proteins [17]. The Gq/11 G proteins are membrane bound GTPases that are linked to 7-TM receptors and are formed by an alpha-, beta- and gamma- subunit [18]. In its inactive form, GDP is bound to the alpha subunit; when catecholamine binds to the receptor it causes a conformational change, which is recognized by the inactive form of the G protein complex and binds to it. The receptor triggers the exchange of bound GDP for GTP on the alpha subunit of the G-protein, which induces the GTP-alpha subunit to dissociate from the beta and gamma subunits. The GTP-alpha subunit (active form) then associates to downstream proteins involved in second messenger signaling cascades [18].

**Fig. 3:**
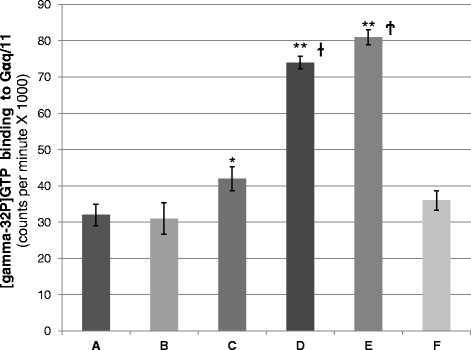
eIF3f promotes adrenoceptor activity upon catecholamine stimulation. Adrenoceptor activity is measured on the basis of *in vitro* agonist-promoted binding of [gamma-32P]GTP to G protein alpha subunits [16], in the presence of cell membrane fractions, and isolated subsequently by immunoprecipitation. In each condition, Gαq/11 immunoprecipitates were analyzed by scintillation for radiolabeled GTP binding. **a** Standard Gαq/11 activity reaction without catecholamine stimulation. **b** Standard Gαq/11 activity reaction with catecholamine stimulation. **c** Gαq/11 activity reaction without catecholamine stimulation and added eIF3f protein. **d** Gαq/11 activity reaction with catecholamine stimulation and added eIF3f protein. **e** Gαq/11 activity reaction with catecholamine stimulation and added eIF3f protein, using membrane fractions obtained from alpha 1B-ADR over-expressed A549 cultures. **f** Standard Gαq/11 activity reaction with catecholamine stimulation, using membrane fractions obtained from over-expressed alpha 1B-ADR A549 cultures. Data and error bars represent means ± standard error (S.E.) for three independent experiments; *p < 0.05 and **p < 0.005 with respect to the controls (A and F), ^ɫ^ *p* < 0.005 between C and D, and ^Ϯ^ *p* < 0.005 between D and E

To determine adrenoceptor activity, *in vitro* experiments were performed using radiolabeled [gamma-32P]GTP, and its transfer to Gαq/11 upon catecholamine stimulation, with or without the presence of eIF3f protein, and with or without over expressed alpha 1B-ADR membranes from A549. Reticulocyte-based translated eIF3f protein was added where indicated or the reticulocyte extract alone where eIF3f protein was not present. Individual assays were further distinctively immunoprecipitated with a Gαq/11 antibody and the [gamma-32P]GTP binding to Gαq/11 was quantified. This assay was based on a previous report [16], where the selectivity and significance in coupling of receptor to GTP-binding regulatory proteins (receptor activation) was demonstrated. Figure 3 shows that the presence of eIF3f promotes adrenoceptor activation.

### The proline- and alanine-rich N-terminal region of eIF3f is not essential for adrenoceptor activation

**Fig. 4:**
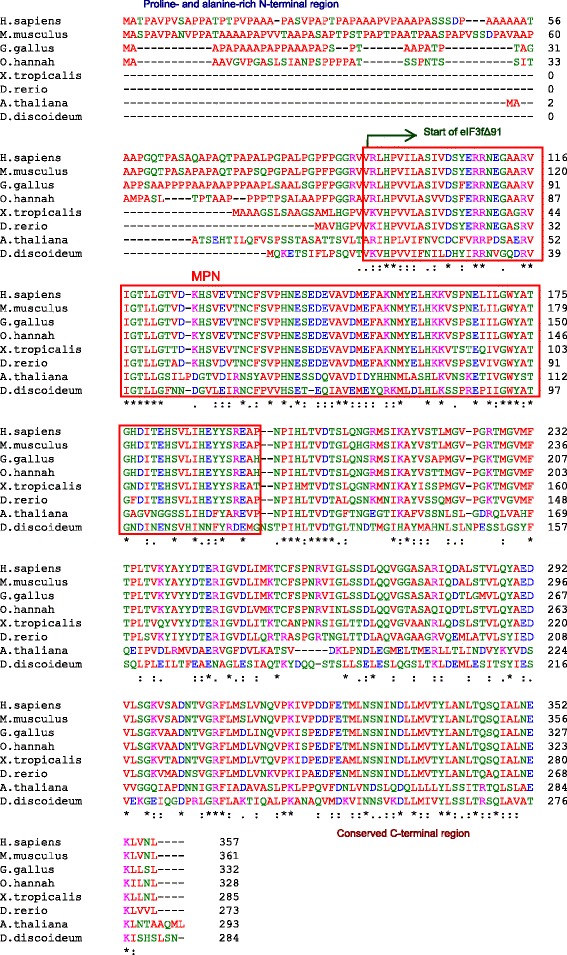
Protein sequence alignment of eIF3f orthologues. eIf3f sequences were obtained from NCBI (*Homo sapiens* gi:6685511, *Mus musculus* gi:341940488, *Gallus gallus* gi: 50749406, *Ophiophagus hannah* gi:565315948, *Xenopus tropicalis* gi:62859127, *Danio rerio* gi:317108137, *Arabidopsis thaliana* gi:23396614, *Dictyostelium discoideum* gi:74850733); and Clustal Omega (EMBL-EBI) was used for multiple sequence alignment. The red box represents the MPN domain. The proline- and alanine-rich N-terminal is present in mammals, non-avian reptiles, and birds, but not in amphibians or lower organisms

**Fig. 5:**
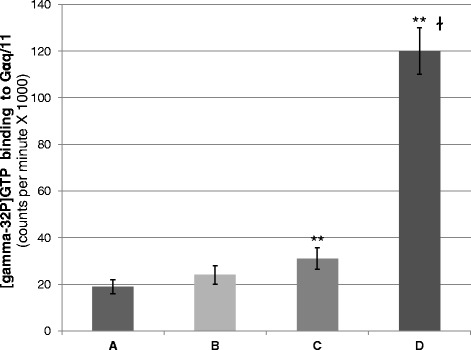
The proline-alanine rich amino terminal of eIF3f is not essential for adrenoceptor activation. The experimental conditions were the same as in Fig. 3. eIF3fΔ91 is a genetic construct where the first 91 amino acids of eIF3f were removed (proline- and alanine-rich N-terminal region). **a** Standard Gαq/11 activity reaction without catecholamine stimulation. **b** Standard Gαq/11 activity reaction with catecholamine stimulation. **c** Gαq/11 activity reaction without catecholamine stimulation and added eIF3fΔ91 protein. **d** Gαq/11 activity reaction with catecholamine stimulation and added eIF3fΔ91 protein. Data and error bars represent mean ± S.E. for three independent experiments; ** *p* < 0.005 with respect to the control (**a**), and ^ɫ^ *p* < 0.0005 between C and D

eIF3f appeared early in Eukaryota, being identified as early as in protists [1]. eIF3f is present in most eukaryotic organisms, except in the budding yeast *Saccharomyces cerevisiae* [1, 6]. All eIF3fs reported to date include the MPN domain [1, 8–10] and a relatively conserved C-terminal region (Fig. 4). However, by comparing protein sequence alignments of eIF3f from the N-terminal region to the beginning of the MPN domain (Fig. 4), it is evident that the proline- and alanine-rich N-terminal region of human eIF3f is a relatively recent evolutionary acquired region. Figure 4 shows the eIF3f protein alignment of some selected organisms. The proline- and alanine-rich N-terminal is present in mammals, non-avian reptiles, and birds, but not in amphibians or lower organisms, which suggests that this region was possibly acquired during the amniote clade. The proline- and alanine-rich N-terminal region of eIF3f has been reported to be important in the functional relationship between eIF3f and other proteins (see introduction), so we then asked if this N-terminal region could be relevant for adrenoceptor activation. To answer this question, we constructed an eIF3f clone that was devoid of this N-terminal region (indicated in Fig. 4), and tested *in vitro* for its ability to promote adrenoceptor activity upon catecholamine stimulation. Figure 5 clearly shows that the N-terminal region of eIF3f is not essential for adrenoceptor activation.

## Discussion

As pointed out earlier, eIF3f has a remarkable ability to interact with many proteins involved in a variety of cellular functions [5, 8–15]. In this work, we report that in native undisturbed conditions, eIF3f stably interacts with the alpha 1B-adrenergic receptor (alpha 1B-ADR) and prompts Gαq/11 activation upon catecholamine stimulation. Previously, eIF3f was located in different subcellular compartments, including the plasma membrane fraction [19], which suggested a different function for eIF3f in addition to the protein synthesis process. Protein immunodetection of eIF3f in native conditions showed a clear single band in A549, HepG2, Ramos, and MC3T3-E1 cells, which localized approximately in the 120 kDa region. Specific immunoprecipitation with anti-eIF3f resolved two clearly distinguished protein bands, a 47 kDa protein corresponding to eIF3f and a 57 kDa protein that, by protein sequencing and specific immunodetection (Fig. 2), was identified as the alpha 1B-ADR. Interestingly, in native conditions we found no free unbound eIF3f protein, which confirms its high ability to interact with other proteins. In fact, most eIF3f is found in the eIF3 complex (data not shown).

In the presence of catecholamine, eIF3f stimulates adrenoceptor activity. The eIF3f/ alpha 1B-ADR interaction represents a novel and fascinating event in the control of adrenoceptor transducing activity, and disclose the possibility of new insights regarding alpha 1B-ADR function in different cellular processes. Our results demonstrate that the proline- and alanine-rich N-terminal region of eIF3f is not required for adrenoceptor activation. Moreover, comparing the GTP binding to Gqα/11 in the presence of native eIF3f (Fig. 3 e) or truncated eIF3f (Fig. 5 D) under catecholamine stimulation, we observed that truncated eIF3f stimulates more GTP binding. Our interpretation is that the N-terminal region of eIF3f lowers its affinity for the adrenoceptor, possibly due to a steric impediment. Other studies should be conducted to elucidate if this has a functional cellular consequence. According to reported eIF3f amino acid sequences, this N-terminal region appeared during the amniote clade (Fig. 4). This was relevant to investigate, since this region was found important for other eIF3f-protein interactions [13, 14]. Taking in account that alpha 1B-ADR is present in vertebrates (Blastp in NCBI and UniProtKB EMBL-EBI) it is possible that the eIF3f/ alpha 1B-ADR interaction would have arisen since then.

To explain the possible cellular function(s) derived from the eIF3f/alpha 1B-ADR interaction, we considered the following facts. The plasma membrane is an organized biological system that serves as a structural barrier and communication interface with the extracellular environment, and the alpha1-adrenergic receptors are embedded in this membrane. Alpha 1- adrenergic receptors bind to and are activated by endogenous catecholamine hormones, which are mainly involved in vasoconstriction [17]. They are coupled to phospholipase C, c-Jun N-terminal kinase, and the mitogen-activated protein kinase downstream signal transduction pathways [17, 20, 21]; and have an important function in stress response that affects lipid, carbohydrate, and amino acid metabolism [22]. In addition to these effects, there is substantial evidence indicating that stimulation of alpha 1-ADRs by catecholamines generally enhances growth-related gene expression and cell growth in a variety of cells, including cardiac myocytes [23], vascular smooth muscle cells [24–26], hepatocytes [20, 27], and adipocytes [28].

Alpha 1B-ADR mediates co-mitogenic effects with catecholamines in different cells. For instance, activation of alpha 1B-ADR increases DNA synthesis in primary cultures of hepatocytes [27], and promotes malignancy in alpha 1B-ADR transfected Rat-1 fibroblasts [29, 30]. Induction of neoplastic transformation by the alpha 1B-ADR, thus, identifies this normal cellular gene as a proto-oncogene. Also, in Rat-1 fibroblasts, alpha 1-ADRs affect the expression of cell cycle-related genes in a differential manner: the over expression of alpha 1A-ADR and alpha 1D-ADR downregulated genes ascribed to the G1/S transition phase, such as Cyclin E and DNA polymerase; this over expression upregulated p27 kip and induced G1/S cell cycle arrest. In contrast, over expressed alpha 1B-ADR transfected cells did not affect Cyclin E or DNA polymerase expression; they showed downregulated p27 kip and stimulated cell cycle progression [30]. On the contrary, CHO cells over expressing human alpha1-adrenergic receptors showed that upon catecholamine activation, alpha 1A-ADR or alpha 1B-ADR -transfected cells exhibit inhibition of serum-promoted cell proliferation and were arrested at G1/S phase, whereas alpha 1D-ADR did not show any effect [31].

eIF3f is also a cell division and proliferation-related gene. In human A549 cells, eIF3f exhibits a fluctuating expression pattern in cycling cells, with maximum expression peaks in G1/S and in G2/M; transient expression analysis showed that eIF3f deregulation compromises cell viability and induces apoptosis [7]. In addition, altered expression of eIF3f has been reported in several human tumors, being found downregulated in some cell lines [32, 33] and upregulated in others [19].

The fact that eIF3f expression is induced in G1/S, that alpha 1B-ADR affects functions related to G1/S, and that both gene products relate physically and functionally, establish the interesting possibility that this relationship might be involved in the regulation of G1/S functions. We are presently exploring this possibility. In addition, since alpha 1B-ADR is considered as a proto-oncogene [29], and the deregulation of eIF3f is frequently associated to oncogenesis, it would be interesting to investigate if and how the interaction of these two proteins affects the control of cell proliferation, and eventually use these gene products as potential targets for cancer therapy.

## Conclusions

In the present work, we report that eIF3f physically and stably interacts with the alpha 1B-ADR, and that eIF3f stimulates adrenoceptor activity. This novel protein-protein interaction may represent a regulatory link between adrenoceptor-related signal transduction and, for instance, cell proliferation and protein synthesis control.

## Abbreviations

alpha 1-ADRs: alpha 1-adrenergic receptors
alpha 1B-ADR: alpha 1B-adrenergic receptor
eIF3f: eukaryotic translation initiation factor 3f
